# Risk factors associated with developmental abnormalities among high-risk children attended at a multidisciplinary clinic

**DOI:** 10.1590/S1516-31802008000100002

**Published:** 2008-01-03

**Authors:** Rosa Resegue, Rosana Fiorini Puccini, Edina Mariko Koga da Silva

**Keywords:** Infant development, Risk factors, Early intervention (Education), Primary health care, Infant, low birth weight, Desenvolvimento infantil, Fatores de risco, Intervenção precoce (Educação), Atenção primária à saúde, Baixo peso ao nascer

## Abstract

**CONTEXT AND OBJECTIVE:**

Knowledge of risk factors associated with child development disorders is essential for delivering high-quality childcare. The objective here was to evaluate the relationships between risk factors and occurrences of developmental abnormalities among children attended at a reference clinic for children at risk of developmental abnormalities.

**DESIGN AND SETTING:**

Retrospective study at a multidisciplinary reference center, Embu, São Paulo.

**METHODS:**

All cases followed up for more than three months between 1995 and 2003 were reviewed. The risk factors assessed were low birth weight, gestational age, length of stay in neonatal ward, perinatal asphyxia, mother's age < 18 years, congenital infections, malformations and low mother's education level. Developmental abnormalities were defined according to developmental tests and assessments by the clinic's professionals. The statistical analysis consisted of the chi-squared test for comparing categorical variables and a logistic regression model for multivariate analysis.

**RESULTS:**

211 children were followed up for more than three months. Developmental abnormalities occurred in 111 (52.6%). Univariate analysis showed significant relationships between developmental abnormality and low birth weight, perinatal asphyxia, length of stay > 5 days, prematurity and mother's age 18 years and older. Low birth weight, history of perinatal asphyxia and mother's age continued to be significant in multivariate analysis.

**CONCLUSIONS:**

Special attention must be paid to the development of low birth weight infants and/or infants with histories of neonatal complications. Low birth weight is easily assessed and should be considered to be an important marker when defining guidelines for following up child development.

## RESUMO

**CONTEXTO E OBJETIVOS:**

O conhecimento dos fatores de risco associados a distúrbios do desenvolvimento é fundamental para a qualidade dos cuidados prestados à criança. O objetivo deste estudo foi analisar a associação entre fatores de risco para atraso de desenvolvimento e as alterações observadas em crianças acompanhadas num ambulatório multidisciplinar.

**TIPO DE ESTUDO E LOCAL:**

Estudo retrospectivo desenvolvido em ambulatório multidisciplinar de referência, Embu, São Paulo, Brasil.

**MÉTODO:**

Revisaram todos os prontuários das crianças acompanhadas por mais de três meses entre 1995 e 2003. Fatores de risco analisados: baixo peso ao nascer, idade gestacional, tempo de permanência no berçário, asfixia perinatal, idade materna < 18 anos, infecção congênita, malformação e baixa escolaridade materna. A alteração do desenvolvimento foi definida pela realização de testes padronizados e pela avaliação dos profissionais envolvidos. Análise estatística: na comparação das variáveis categóricas, utilizou-se o Teste Qui-Quadrado. Para análise multivariada, utilizou-se o modelo de regressão logística.

**RESULTADOS:**

211 crianças foram acompanhadas por mais de três meses. Alterações do desenvolvimento ocorreram em 111 crianças (52,6%). Na análise univariada, baixo peso ao nascer, asfixia perinatal, tempo de permanência no berçário > 5 dias, prematuridade e mães > 18 anos apresentaram relação significante com alterações do desenvolvimento. Na análise multivariada, asfixia perinatal, baixo peso ao nascer e idade materna mantiveram essa associação.

**CONCLUSÕES:**

Especial atenção deve ser dada ao desenvolvimento das crianças com baixo peso ao nascer e/ou história de intercorrências neonatais. O baixo peso ao nascer deve ser considerado importante marcador ao definir-se diretrizes para o acompanhamento do desenvolvimento das crianças.

## INTRODUCTION

Over recent years, changes in childhood morbidity-mortality patterns, decreased fertility of the population and, particularly, new findings regarding how the brain functions during the first years of life have led to increased interest in child development. Knowledge of the risk factors associated with disturbances in the child development process is essential for delivering high quality child care.^1^

Risk factors for developmental problems have been divided into environmental and biological categories, although factors from these two categories are often synergistic. Several environmental factors may influence an individual's development, such as interpersonal relationships developed in specific contexts; different scenarios to which individuals belong at specific periods of their lifetimes; social structures relating to group cultural values; and the individual's political, historical and economic times.^2,3^ The biological factors include prematurity, perinatal asphyxia, peri-intraventricular hemorrhage, bronchopulmonary dysplasia, biochemical and hematological disorders during the neonatal period, microcephaly, malformations, congenital infections and low birth weight.^4–6^

There has been great improvement in the survival of preterm infants over the last three decades, following the implementation of neonatal intensive care units.^6^ However, improvements in survival rates among these infants have not had any homogeneous impact with regard to reducing sequelae during development. There is still much concern regarding short, medium and long-term prognosis, particularly for extremely premature infants (gestational age < 26 weeks), very low birth weight infants (birth weight < 1500 g) and extremely low birth weight infants (birth weight < 1000 g).^7^ The prognosis for the development of preterm infants appears to be directly related to the presence of other conditions such as bronchopulmonary dysplasia, retinopathy of prematurity and intracranial changes on ultrasound, which significantly worsen the prognosis of these infants.^8^

Low birth weight has been considered to be an important risk factor for child morbidity and mortality, and also a socioeconomic development indicator for geographic regions. However, recently conducted population studies have demonstrated a change in this paradigm, showing that the prevalence of low birth weight can no longer be considered to be a pivotal indicator for social development.^9^ Differences in access to and quality of perinatal care, which have resulted in increased survival of low birth weight preterm infants that previously had been considered to be fetal deaths, could be some of the factors leading to this change.

Early detection and intervention among children with developmental disorders are important prognostic factors for quality of life.^10,11^ Therefore, there is concern with regard to developing a healthcare model capable of encompassing this entire process, taking into account the sociocultural differences among children and also identifying risk factors in order to establish priorities for the actions to be taken.^12,13^ In this light, in 1995 a reference multidisciplinary outpatient clinic was created as part of the primary healthcare program of the municipality of Embu, State of São Paulo, Brazil, with the aim of providing follow-up for children at risk of developmental abnormalities. Comprehensive criteria that could be easily be identified by healthcare professionals and that, when present, would lead to other existing factors were proposed for inclusion in the program.

## OBJECTIVE

The objective of this study was to evaluate the relationship between presence of risk factors and findings of developmental abnormalities during follow-up among children attending a reference multidisciplinary outpatient clinic, with the aim of providing follow-up for children at risk of developmental abnormalities.

## METHODS

This was a retrospective study to evaluate all the children enrolled in the program between 1995 and 2003 who were followed up for at least three months. The risk factors defined for referral to the outpatient clinic included low birth weight (< 2500 g), length of stay in neonatal ward > 5 days (since this is the maximum number of days of hospitalization needed for women undergoing the cesarean procedure), mothers under 18 years of age, history of perinatal asphyxia, presence of congenital malformations, congenital infection and history of maternal drug abuse.

Because of the importance of gestational age and the mother's education level, we decided that these issues would also be assessed, although they had not been included in the criteria for referral to the program.^5,14^ Prematurity was defined as less than 37 weeks of gestation, while perinatal asphyxia was defined as an Apgar score of 0 to 3 at 5 minutes, umbilical artery pH < 7.0, signs of neurological complications and signs of multisystem organ dysfunction during stay in neonatal ward.^15^ In cases in which data were not available, particularly among preterm infants and because of the poor reliability of the Apgar score, perinatal asphyxia was diagnosed when the discharge summary indicated a suggestive clinical outcome (signs of multisystem organ dysfunction and/or neurological complications).

The diagnosis was based on the Denver developmental screening test,^16^ specific evaluation of hearing, motor, language and visual functions and neurological evaluation. The final diagnosis was made from the evaluations by the healthcare professionals at the outpatient clinic (one pediatrician, two speech therapists, one physiotherapist, one psychiatrist and one neuropediatrician). Abnormalities recorded in at least two evaluations were included in the analysis.

Hearing assessment was performed through observation of the behavioral response to sound stimuli, visual reinforcement audiometry (in children > 6 months of age), observation of responses to verbal stimuli and middle ear evaluation by otoscopy performed by the pediatrician, as proposed by Azevedo.^17^ This assessment allowed the child's hearing capabilities to be registered and classified into one of the following categories: normal; delayed development (when responses were below the expected standard, but later returned to normal); and development disorder (when responses remained below the normal expected standard). In this study, suspected deficit was also included when the child presented abnormal responses or no response. After the year 2000, when the appropriate equipment was acquired, all children with birth weight under 1500 g admitted to the outpatient clinic underwent otoacoustic emission examination before discharge from the referral neonatal ward of the region. Language assessment was performed using the behavioral observation protocol for children aged 0-6 years.^18^

Motor assessment was performed through neurological evaluation, consisting of posture observations with the child in the prone, supine, seating or standing positions, and also circular reactions. The neurological examinations were performed by an experienced, certified neurologist.

The visual assessment was performed using the preferential looking technique^19^ and fundoscopy.

### Statistical analysis

The chi-squared test was used for comparing categorical variables, using the Epitable function in the Epi-Info 6.01 software^20^ for the calculations. A logistic regression model was used for multivariate analysis, using the MULTLR software.^21^ A 5% significance level was used for the inclusion of independent variables in the logistic regression model. A 5% significance level was also used in all other statistical tests (alpha = 0.05). When the calculated p value (minimum level of significance) allowed rejection of the null hypothesis, this was indicated in bold (p < 0.05). This research project was approved by the Ethics Committee of Universidade Federal de São Paulo (Unifesp)

## RESULTS

Over the study period, 262 children were enrolled in the program, and 211 (80.5%) were followed up for at least three months. As shown in Table 1, length of stay in the neonatal ward and low birth weight were the most frequent risk factors. Histories of unstructured families, meningitis during the first year of life, mother or father with psychiatric problems, death of the mother and child conceived as the result of rape were the referral factors for 10 children who were admitted for other reasons.

**Table 1.** Type of risk among children enrolled for follow-up due to risk of developmental delay. Municipality of Embu, 1995-2003

Type of risk	n	%*
Length of stay in neonatal ward > 5 days	146	69.2
Birth weight < 2500 g	117	55.5
Perinatal asphyxia	70	33.2
Mother ≤ 18 years of age	62	29.4
Gross malformation	5	2.4
Congenital infection	5	2.4
Other	10	4.7

* Percentage calculated based on 211 children, who could present one or more risk factors each.

Table 2 shows some sociodemographic and childbirth-related features of the children followed up. Out of the total of 211 children, 104 (49.3%) were born preterm and 107 (50.7%) were born at full term. Most mothers of the referred children had attended school for up to eight years.

**Table 2.** Sociodemographic and childbirth-related features of children followed up for more than 3 months. Municipality of Embu, 1995-2003

	n	%
**Gender**		
Male	103	48.8
Female	108	51.2
**Age at beginning of follow-up**		
< 6 months	171	81.0
6-11 months	35	16.6
≥12 months	5	2.4
**Prenatal care** *		
Yes	197	93.4
No	14	6.6
**Type of delivery**		
Natural	111	52.6
Cesarean	91	43.1
Forceps	9	4.3
**Birth weight (g)**		
< 1000	8	3.8
1000 – 1499	33	15.6
1500 – 2499	76	36.0
2500 – 2999	46	21.8
≥ 3000	48	22.7
**Gestational age**		
Full term	107	50.7
Preterm	104	49.3
**Mother's education level** †		
< 4 years	47	22.5
5-8 years	108	51.7
> 8 years	54	25.8
**Father's education level** ‡		
< 4 years	61	32.3
5-8 years	95	50.3
> 8 years	33	17.4
**Mother's age** §		
< 18 years	61	29.0
18 – 20	19	9.0
20 – 30	81	38.6
30 – 40	43	20.5
≥ 40	6	2.9

* No information available = 3 (0.6%);

† No information available = 2 (0.9%);

‡ No information available = 22 (11.6%);

§ No information available = 1 (0.5%).

During the follow-up period, 111 children (52.6%) presented developmental abnormalities and the most frequent of these were hearing, motor and language impairment (Table 3).

**Table 3.** Type of abnormality observed among children at risk of developmental abnormalities followed up for more than three months. Municipality of Embu, 1995-2003

Type of abnormality	n	%*
Hearing	81	73.0
Motor function	53	47.7
Language	34	30.6
Visual	13	11.7
Morphological	9	8.1
Mother-child relationship	7	6.3
Behavior	3	2.7
Other	4	4.5

* Percentage calculated based on 111 children.

The hearing abnormalities observed included delayed hearing development, which was seen in 33 children (40.8%); absence of cochleopalpebral reflex in 30 (37.0%); development disorder in 12 (14.8%); and suggestive findings of deficit in six (7.4%) children. Delayed motor acquisition and tone abnormalities were the most frequent motor abnormalities, seen in 83% and 45.3% respectively of the total of 53 children with motor abnormalities. Delayed language acquisition was seen in 34 children who presented this type of impairment.

Among the 13 children who presented visual abnormalities, eight (61.5%) had strabismus, two (15.4%) had refraction errors, three (23.1%) had retinopathy of prematurity and one (7.7%) had eye fundus changes without a definite diagnosis. The following morphological changes were observed: craniostenosis, cranial asymmetry, microcephaly, significantly increased cephalic perimeter, phenotypic deviation, cleft palate and harelip. Problems in mother-child relationships were seen in seven cases: three mothers were depressed, three mothers overprotected their children due to a fear of losing them and one mother had had an unwanted pregnancy. The behavioral abnormalities seen included exacerbated aggressive behavior, night terror and passive behavior with stereotypical rocking movements. Other abnormalities included seizures in two children, delayed social development in one child and school difficulties in one child.

Comparing the frequencies of risk factors between children with and without any developmental abnormalities, univariate analysis showed statistically significant relationships with birth weight under 2500 g, occurrence of perinatal asphyxia, length of stay in neonatal ward longer than five days, prematurity and mother's age > 18 years (Table 4). Multivariate analysis for any development abnormalities as outcomes showed that these risk factors remained significant, except for duration of hospital stay and prematurity (that were left out of Table 5). These factors were correlated with birth weight. All other possible interactions between the variables were analyzed and did not show statistical significance.

**Table 4:** Presence of developmental abnormalities observed among children followed up for more than three months, according to risk factors. Municipality of Embu, 1995-2003

Risk factors	Developmental abnormality		No change		Total		p-value
	n	%	n	%	n	%	
**Mother's age < 18 years**							
Yes	17	27.4	45	72.6	62	29.4	**< 0.01**
No	94	63.1	55	36.9	149	70.6	
**Perinatal asphyxia**							
Yes	54	77.1	16	22.9	70	33.2	**< 0.01**
No	57	40.4	84	59.6	141	66.8	
**Birth weight**							
< 1500 g	32	78.0	9	22.0	41	19.4	**< 0.01**
1500-2500 g	45	59.2	31	40.8	76	36.0	
> = 2500 g	34	36.2	60	63.8	94	44.6	
**Length of stay in neonatal ward > 5 days**							
Yes	95	65.1	51	34.9	146	69.2	**< 0.01**
No	16	24.6	49	75.4	65	30.8	
**Preterm infant**							
Yes	67	64.4	37	35.6	104	49.3	**< 0.01**
No	44	41.1	63	58.9	107	50.7	
**Malformations**							
Yes	4	80.0	1	20.0	5	2.4	0.37
No	107	51.9	99	48.1	206	97.6	
**Neonatal infection**							
Yes	1	20.0	4	80.0	5	2.4	0.19
No	110	53.2	94	46.8	206	97.6	
**Other risk factors**							
Yes	4	40.0	6	60.0	10	4.7	0.52
No	107	53.2	94	46.8	201	95.3	
**Mother's education level**							
< 4 years	26	55.3	21	44.7	47	22.3	0.44
5 to 8 years	53	49.1	55	50.9	108	51.2	
> 8 years	32	59.3	22	40.7	54	25.6	

**Table 5.** Results from logistic regression analysis for the presence of developmental abnormalities among children followed up for more than 3 months. Municipality of Embu, 1995-2003

Variable	Coefficient	p-value	Odds ratio (OR)	Confidence interval
Mother's age ≤ 18 years	-0.84	0.03	0.43	0.20-0.91
Perinatal asphyxia	1.31	< 0.01	3.71	1.81-7.63
Birth weight < 1500 g	1.02	0.03	2.78	1.07-7.20
Birth weight 1500-2500 g	0.77	0.03	2.17	1.07-4.40

Regarding the final diagnoses of abnormalities among the children, 17 (15.3%) out of 111 presented with permanent abnormalities: 11 with cerebral palsy, two with retinopathy of prematurity (one with severe visual impairment), one with West Syndrome, one with epilepsy and two with craniostenosis.

Among the 11 children with cerebral palsy, six were preterm and progressed with cerebral diplegia or hemiplegia. Out of the five children born at full term, four had histories of perinatal asphyxia and one of congenital infection. Four of these children progressed with spastic quadriplegia and one with hemiplegia. Abnormalities in hearing development were seen in 10 children (90.9%), and a diagnosis of hearing deficit was made for four children. Visual abnormalities were present in six (54.5%) of the 11 children. Delayed language development was seen in all children, and five (45.4%) had no speech development.

## DISCUSSION

Among the selected risk factors for enrollment of children in the outpatient clinic in the current study, low birth weight showed significant correlation with developmental abnormalities, both in univariate and multivariate analyses.

Low birth weight is related to the duration of gestation and to the fetal growth pattern. Therefore, low weight can be attributed to prematurity or intrauterine growth retardation (IGR), and such children form a heterogeneous group that requires different intervention strategies. In Brazil, data from the 1990s indicated similar proportions of low birth weight due to prematurity and to IGR. However, there was evidence of increased frequency of preterm labor.^22^ Trials carried out in two Brazilian cities, i.e. Pelotas (State of Rio Grande do Sul) and Ribeirão Preto (State of São Paulo), confirmed this tendency.^23,24^

Historically, studies conducted to evaluate outcomes among low birth weight infants have focused on the relationship between low birth weight and morbidity-mortality during childhood.^9^ Over recent years, trials focused on evaluating the prognosis for child development have generally been conducted in developed countries and have included infants with birth weight under 1500 g, and particularly those with less than 32 weeks of gestation.^25–27^ However, whether low birth weight is due to prematurity or IGR, it constitutes a risk factor for developmental abnormalities. This association is particularly frequent under unfavorable social conditions. In these situations, social risk factors such as the parents’ education level, unstructured families and psychiatric problems in the family are not isolated occurrences but, rather, cyclic determining conditions. The combination of several factors results in an increased likelihood of developmental abnormalities, which amplifies biological vulnerability.^1,2,28^ Studies conducted both in the northeastern and southern regions of Brazil have corroborated the existence of a relationship between low birth weight and developmental abnormalities. Grantham-McGregor et al.^29^ carried out a one-year follow-up study among children from a city in northeastern Brazil: 131 born at full term with birth weight between 1500-2499 g and 131 children with birth weight between 3000 g and 3499 g. The low birth weight infants presented decreased development at six and twelve months and higher vulnerability to social variables such as the mother's education level and home stimuli. Halpern et al.^30^ showed in a study conducted in southern Brazil that low birth weight children had a threefold greater chance of presenting developmental abnormalities at 12 months of age.

Regarding the length of stay in the neonatal ward, the association between this factor and developmental abnormalities was not assessed in the multivariate analysis probably because of the heterogeneity of the group, which included children admitted to treat mild conditions and others admitted for longer periods, usually in the intensive care unit.

Perinatal asphyxia occurred in 96 (35.6%) of the children referred to the outpatient clinic. Historically, perinatal cerebral damage was considered to be a major cause of cerebral palsy and mental impairment. However, this assumption has been demystified in recent studies that have indicated that only the most severe and long-lasting cases of asphyxia can cause these abnormalities.^31^ In the present study, perinatal asphyxia was related to developmental abnormalities, whether analyzed jointly or separately, both in the univariate and in the multivariate analysis. However, it is worth remembering that histories suggestive of asphyxia recorded in the hospital discharge summary were considered to be perinatal asphyxia in this study, particularly in cases of preterm births. This information is not always easily obtainable in most public healthcare services.

One of the criteria selected for referring infants to the outpatient clinic was mother's age < 18 years. The selection of this age group, differing from the guidelines proposed by the World Health Organization (WHO), which define adolescence as the period of life between 10 and 20 years of age, was based on data from the literature that give evidence for lower occurrence of pregnancy problems among women aged 18 years and above.^32^ In the present study children with this risk factor did not show developmental abnormalities. Initial studies on teenager pregnancy indicated that pregnancy during adolescence was an important risk factor for low birth weight, preterm labor, infant mortality and developmental abnormalities.^32^ However, there is evidence that these outcomes are related to other variables such as socioeconomic condition, community support and quality of health service, which also favor pregnancy in this group age. The harmful effects of pregnancy during adolescence would be more evident in pregnant girls under 15 years of age.^32^

Regarding the final outcomes for the children followed up in this service, it is difficult to compare our results with data from other studies, since most authors used preterm follow-up cohorts from outpatient clinics, hospitalized babies or babies from wards in several institutions. In this type of outpatient clinic, the major concern is frequently to assess the prognosis. Moreover, these services are not usually integrated with the local healthcare network. Public policy discussions focusing on this group of children are recent, and there are few reports on experiences of implementing regional and hierarchical healthcare models, either for infants at high risk of developmental abnormalities or infants with established abnormalities.

## CONCLUSIONS

Special attention must be paid to the development of low birth weight infants and infants with neonatal complications. Since low birth weight can easily be determined, it should be considered to be an important marker when defining guidelines for infant development care programs.

## References

[1] Walker SP, Wachs TD, Gardner JM (2007). Child development: risk factors for adverse outcomes in developing countries. Lancet.

[2] Sameroff AJ (1998). Environmental risk factors in infancy. Pediatrics.

[3] Lordelo ER, Lordelo ER, Carvalho AMA, Koller SH (2002). Contexto e desenvolvimento humano: quadro conceitual. Infância brasileira e contextos de desenvolvimento.

[4] Platt MJ, Cans C, Johnson A (2007). Trends in cerebral palsy among infants of very low birthweight (<1500 g) or born prematurely (<32 weeks) in 16 European centres: a database study. Lancet.

[5] Patra K, Wilson-Costello D, Taylor HG, Mercuri-Minich N, Hack M (2006). Grades I-II intraventricular hemorrhage in extremely low birth weight infants: effects on neurodevelopment. J Pediatr.

[6] Hack M, Taylor HG (2000). Perinatal brain injury in preterm infants and later neurobehavioral function. JAMA.

[7] Wood NS, Marlow N, Costeloe K, Gibson AT, Wilkinson AR (2000). Neurologic and developmental disability after extremely preterm birth. EPICure Study Group. N Engl J Med.

[8] Aylward GP (2003). Cognitive function in preterm infants: no simple answers. JAMA.

[9] da Silva AA, Bettiol H, Barbieri MA (2003). Infant mortality and low birth weight in cities of Northeastern and Southeastern Brazil. Rev Saude Publica.

[10] Grantham-McGregor S, Cheung YB, Cueto S (2007). Developmental potential in the first 5 years for children in developing countries. Lancet.

[11] Hernandez Muela S, Mulas F, Mattos L (2004). Plasticidad neuronal functional. [Functional neuronal plasticity]. Rev Neurol.

[12] (2001). Developmental surveillance and screening of infants and young children. Pediatrics.

[13] Köhler L, Rigby M (2003). Indicators of children's development: considerations when constructing a set of national Child Health Indicators for the European Union. Child Care Health Dev.

[14] National Institute of Child Health and Human Development Early Child Care Research Network (2005). Duration and developmental timing of poverty and children's cognitive and social development from birth through third grade. Child Dev.

[15] Santos AMN, Ferlin MLS, Rugolo LMSS (2000). Asfixia perinatal. Manual de Neonatologia. Sociedade de Pediatria de São Paulo.

[16] Frankenburg WK, Dodds JB (1967). The Denver developmental screening test. J Pediatr.

[17] Azevedo MF (1991). Avaliação e acompanhamento audiológico de neonatos de risco. [Audiological assessment and follow-up programme for neonates at risk]. Acta AWHO.

[18] Chiari BM, Basílio CS, Nakagwa EA (1991). Proposta de sistematização de dados da avaliação fonoaudiológica através da observação de comportamentos de crianças de 0 a 6 anos [Proposal of data systematization of the phonoaudiological evaluation by observation of children's behavior from 0 to 6 years old]. Pró-Fono R Atual Cient.

[19] Teller DY, McDonald MA, Preston K, Sebris SL, Dobson V (1986). Assessment of visual acuity in infants and children: the acuity card procedure. Dev Med Child Neurol.

[20] Dean AG, Dean JA, Colulombier D (1995). EpiInfo version 6: a word-processing database, and statistics program for public health on IBM-compatible microcomputer.

[21] Campos N, Franco EL (1989). Epidemiologic programs for computers and calculators. A microcomputer program for multiple logistic regression by unconditional and conditional maximum likelihood methods. Am J Epidemiol.

[22] Victora CG, Barros FC (2001). Infant mortality due to perinatal causes in Brazil: trends, regional patterns and possible interventions. Sao Paulo Med J.

[23] Horta BL, Barros FC, Halpern R, Victora CG (1996). Baixo peso ao nascer em duas coortes de base populacional no sul do Brasil. [Low birthweight in two population-basaed cohorts in southern Brazil]. Cad Saúde Pública = Rep Public Health.

[24] Bettiol H, Rona RJ, Chinn S, Goldani M, Barbieri MA (2000). Factors associated with preterm births in southeast Brazil: a comparison of two birth cohorts born 15 years apart. Paediatr Perinat Epidemiol.

[25] Emsley HC, Wardle SP, Sims DG, Chiswick ML, D'Souza SW (1998). Increased survival and deteriorating developmental outcome in 23 to 25 week old gestation infants, 1990-4 compared with 1984-89. Arch Dis Child Fetal Neonatal Ed.

[26] Hack M, Fanaroff AA (1999). Outcomes of children of extremely low birthweight and gestational age in the 1990's. Early Hum Dev.

[27] Hoekstra RE, Ferrara TB, Couser RJ, Payne NR, Connett JE (2004). Survival and long-term neurodevelopmental outcome of extremely premature infants born at 23-26 weeks’ gestational age at a tertiary center. Pediatrics.

[28] de Andraca I, Pino P, de la Parra A, Rivera F, Castilo M (1998). Factores de riesgo para el desarrollo psicomotor em lactantes nacidos en óptimas condiciones biológicas. [Risk factors for psychomotor development among infants born under optimal biological conditions]. Rev Saude Publica.

[29] Grantham-McGregor SM, Lira PI, Ashworth A, Morris SS, Assunção AM (1998). The development of low birth weight term infants and the effects of the environment in northeast Brazil. J Pediatr.

[30] Halpern R, Barros FC, Horta BL, Victora CG (1996). Desenvolvimento neuropsicomotor aos 12 meses de idade em uma coorte de base populacional no sul do Brasil: diferenciais conforme peso ao nascer e renda familiar. [Developmental status at 12 months of age in a cohort of children in southern Brazil: differences according birthweight and family income]. Cad Saúde Públ = Rep Public Health.

[31] Robertson CM, Finer NN (1993). Long-term follow-up of term neonates with perinatal asphyxia. Clin Perinatol.

[32] Mariotoni GGB, Barros AA (1998). A gravidez na adolescência é fator de risco para o baixo peso ao nascer? [Is adolescent pregnancy a risk factor for low birth weight?]. J Pediatr (Rio de J).

